# Supplementary material for: Parrots imitate song and dance with mixed vocal-gestural neural signals
Source: bioRxiv. 2026 Oct 22:2026.10.21.760654. Preprint. [Version 1] doi: 10.64898/2026.10.21.760654 (PMC13738777; doi:10.64898/2026.10.21.760654)
Supplement: Supplementary file 1 [file NIHPP2026.10.21.760654v1-supplement-1.pdf]

bioRxiv preprint doi: https://doi.org/10.64898/2026.10.21.760654; this version posted October 22, 2026. The copyright holder for this preprint (which was not certified by peer review) is the author/funder, who has granted bioRxiv a license to display the preprint in perpetuity. It is made available under a CC-BY 4.0 International license.

Supplementary Materials for

Parrots imitate song and dance with mixed vocal-gestural neural signals

Caleb Jones, Han Kheng Teoh, Julie Carpenter, Andrea Roeser, Ruidong Chen, Brian Kardon, Zhilei Zhao, Itai Cohen and Jesse Goldberg

Corresponding author: jessehgoldberg@gmail.com

The PDF file includes:

Materials and Methods
Supplementary Text
Figs. S1 to S6
Table S1
References

Other Supplementary Materials for this manuscript include the following:

Movies S1 to S8

bioRxiv preprint doi: https://doi.org/10.64898/2026.10.21.760654; this version posted October 22, 2026. The copyright holder for this preprint (which was not certified by peer review) is the author/funder, who has granted bioRxiv a license to display the preprint in perpetuity. It is made available under a CC-BY 4.0 International license.

Materials and Methods

Behavioral recordings and social paradigms

For behavioral experiments, two colonies of budgerigars (*Melopsittacus undulatus*) were housed in visual and acoustic isolation (Cohort A, A1-A4; Cohort B, B1-B4) in 18" x 18" x 31.5" cages. Environments were maintained on a 12h light cycle using 850nm IR LEDs to facilitate infrared video acquisition. Mirror-directed behavior was recorded at 90 fps using an IR-sensitive camera (Flir Blackfly S BFS-U3-32S4M, Kowa LM16FC24M lens, Hoya IR (R72) filter) positioned behind a one-way mirror, while synchronized audio was captured at 50 kHz (Audio-Technica A35). For social re-pairing experiments, birds from both cohorts were initially co-housed in a large group cage for 6 weeks before being transitioned into cross-cohort dyads (one bird from each original colony). Dyadic interactions were recorded 10 months post-pairing. Birds were identified by unique facial characteristics (iris contrast, cheek spots)(*47*), and mirror-directed "interactions" were defined as periods of warbling and gesturing exceeding 1 s.

Warble segmentation

Warble song was segmented and labeled using a fine-tuned WhisperSeg-base transformer model (*14*) trained on approximately 6000 manually labeled syllables from four birds. This model (F1 = 0.878) classified vocalizations into five categories: contact calls, broadband squawks, rhythmic compound syllables, broadband clicks, and G-notes (*10,16*). To quantify acoustic similarity in segmented syllables across individuals, we extracted 11 acoustic features from each syllable spectrogram: the mean and standard deviation of mean frequency, spectral entropy, spectral flatness, spectral skewness, and modulation rate (defined as the first temporal derivative of mean frequency), as well as total syllable duration (*15*). All features were z-score normalized across birds within each syllable label to ensure comparability across feature scales. For each syllable label, we computed pairwise Euclidean distances (d_{ij}) between the standardized feature vectors of all bird pairs. These distances were transformed into similarity scores using the relation $S_{ij} = 1 / (1 + d_{ij})$. These scores were then averaged for each unique pair of birds. To account for individual differences in self-similarity and vocal variability, factors not typically addressed in standard songbird analyses (*15*), we applied a geometric normalization: each similarity score was divided by the square root of the product of the corresponding self-similarity values, $S_{ij} / \sqrt{S_{ii} * S_{jj}}$. We utilized these aggregated, normalized similarity scores to evaluate whether the mean similarity among intra-group pairs differed significantly from inter-group pairs via a permutation test.

Headbob analyses

In the behavioral dataset, head position (cere) was tracked via DeepLabCut (*22*). Trajectories were detrended using a 340-ms median filter and transformed via a Morlet wavelet (scales 1–60). Headbob bouts were identified by spectral power peaks at scales 40–45, with headbob frequency (F) defined as $F = (fc * fs) / a$, where $fc = \pi/3$ and fs is the sampling frequency. Temporal coupling between syllables and headbob phase was quantified via bird-level permutation tests (10,000 iterations), using the squared distance between cohort-mean probability curves as a test statistic. Segmented intervals were then manually reviewed to eliminate potential false positives. To quantify syllable production in headbob bias at the level of individual birds, we computed a preference score (Compound − Click) for each bird. Preference scores were positive for all A birds and negative for all B birds, and differed significantly between cohorts (Mann–Whitney U test, $N_A=4$, $N_B=3$, $p = 0.028$), confirming the cohort

bioRxiv preprint doi: https://doi.org/10.64898/2026.10.21.760654; this version posted October 22, 2026. The copyright holder for this preprint (which was not certified by peer review) is the author/funder, who has granted bioRxiv a license to display the preprint in perpetuity. It is made available under a CC-BY 4.0 International license.

specific vocal gesture coupling. To quantify coupling of syllable timing to headbob phase, we performed a bird-level permutation test in which phase-dependent probability curves were computed for each animal and birds were randomly reassigned to cohorts (10,000 permutations). The squared distance between cohort-mean curves was used as the test statistic, revealing a significant difference between colonies, $p = 0.025$). We performed a bird-level permutation test using a global statistic defined as the sum of per-syllable distances between cohort-mean curves. This omnibus test revealed a strong cohort effect ($p = 9.9\times10^{-5}$). Post hoc syllable-specific permutation tests with Benjamini–Hochberg FDR correction across five categories showed significant cohort differences for call, click, squawk, and G syllables ($q=0.0347$ for each), whereas compound syllables approached significance ($q=0.0576$).

Latent space analyses and predictive networks

To represent fine-scale subsyllabic acoustic structure, we implemented a shotgun Variational Autoencoder (VAE) with a 32D latent space and tanh activations (*20*). Spectrograms were processed using an 80-ms sliding window (5-ms steps) and resized to 32 x 128 pixels. The model was trained for 50 epochs (ADAM optimizer, learning rate 0.001) using a 90/10 train-test split. Cross-bird acoustic similarity was quantified using a reconstruction-based metric. For a VAE trained on a reference bird, mean-squared reconstruction error (MSE) was computed for both training data and evaluation data from held out data or another bird. Similarity was defined as the ratio MSE(train)/MSE(eval), with values approaching 1 indicating high similarity. For supplemental analyses, similarity was also assessed using conventional acoustic features (frequency statistics, entropy, spectral shape, modulation rate, and duration)(*15*). Feature vectors were z-scored within syllable category, pairwise Euclidean distances were computed, and distances were converted to similarity scores. Group differences were evaluated by permutation testing. An LSTM classifier was trained to predict head-bob occurrence based on the preceding 200 ms of VAE latent features. The network consisted of two LSTM layers (hidden size 50) followed by a fully connected output layer. Models were trained per bird (80% training / 20% testing) using cross-entropy loss and evaluated on held-out and cross-bird data. The output log-likelihood was smoothed using a 100 millisecond moving average. The learning rate was 0.01, batch size 20, and the model was trained for 50 epochs. Classifier performance was evaluated on both held-out and cross-bird datasets.

Surgery and neural recording

For chronic neural recordings, 4 birds were anesthetized with isoflurane inhalation and 16-channel moveable wire bundles were implanted into AAC coordinates (+5.85-6.05 A, +5.4 L [relative to lambda], 2.6-3.0 V [relative to pial surface]; head angle 30°). Birds were then placed in dyadic chambers with a partner for post-operative recovery on a 12 hour light/dark cycle with ad libitum food and water. Neural signals were amplified, filtered, and digitized on Intan RHD headstages equipped with 3-axis accelerometers. Headstages were connected to the implanted 16-channel moveable electrode bundles from Innovative Neurophysiology. Electrodes were extended or retracted on a semi-daily basis by manual turning of a screw in the morning before the enclosure lights came on. Neural signals were further band-pass filtered and sorted in a custom MATLAB GUI.

Neural spiking alignment to vocalizations and headbobs

To test if a neuron exhibited a significant response to a given syllable type, spike times were aligned to syllable onset and binned in 10 ms intervals from −0.1 s relative to onset through the median duration of that syllable type. Peri-stimulus time histograms (PSTHs) were computed

bioRxiv preprint doi: https://doi.org/10.64898/2026.10.21.760654; this version posted October 22, 2026. The copyright holder for this preprint (which was not certified by peer review) is the author/funder, who has granted bioRxiv a license to display the preprint in perpetuity. It is made available under a CC-BY 4.0 International license.

by averaging across trials and smoothed using a 3-bin moving average. Headbob-aligned PSTHs were constructed using the same procedure. To assess statistical significance of observed firing rate modulations, we generated a null distribution using circular spike-time shuffling (*48*). For each neuron and syllable type, spike trains were randomly circularly shifted 1000 times, and a shuffled PSTH was computed for each iteration. The maximum and minimum firing rates were extracted from each shuffled PSTH to form null distributions of peak and trough activity. The empirical maximum and minimum firing rates were then compared to these null distributions to compute p-values. A neuron was considered to exhibit a significant vocal response if either the maximum or minimum firing rate exceeded the 95th percentile of the corresponding null distribution ($p < 0.05$). Headbob-aligned PSTHs were constructed using the same procedure. Neurons recorded during at least 20 headbobs with at least 20 spikes during those headbobs were included in the headbob-related analyses in Fig 3.

Headbob influence on syllable-aligned neural activity

For neurons that exhibited significant call-aligned activity, all call syllables were time-warped to the median duration and grouped according to whether they occurred during headbobs (HB) or outside of them. To isolate neural modulation attributable to the HB context, each HB syllable was paired with its nearest acoustic neighbor produced outside of HB (cosine distance < 0.3 in flattened spectrogram space). Only HB syllables with a matched non-HB counterpart were included in the analysis. Time-warped spike trains from HB syllables and their acoustically matched non-HB counterparts were used to construct firing rate histograms for the two conditions. To assess statistical significance of firing rate differences in each 10 ms time bin, syllables and associated spike trains were randomly reassigned pseudo-labels (HB or non-HB), and firing rate histograms were recomputed for 10,000 iterations to generate a null distribution of bin-wise differences. Observed differences were compared against this null distribution. Neurons exhibiting a significant difference ($p<0.01$) in at least 3 consecutive time bins were considered to be HB-modulated.

Statistical analysis of effect of recording depth on neural representations

To assess population-level effects of recording depth on neural activity while accounting for hierarchical data structure, we combined principal component analysis (PCA) with linear mixed-effects modeling (LME) and a within-channel permutation test. Firing rate matrices were first reduced using PCA (units x time bins). PCs explaining at least 95% of the cumulative variance were retained for all subsequent analysis. Each retained PC was weighted by its proportion of explained variance to preserve the contribution of dominant population modes. For each retained PC, we fit a linear mixed-effects model with both linear and quadratic depth terms:

$$PCi \sim Depth + Depth^2 + (1|Bird) + (1|Bird{:}Channel)$$

Depth was mean-centered within-recording channel prior to analysis to isolate within-channel depth structure and eliminate between-channel offsets. Random intercepts for Bird and Bird x Channel accounted for repeated measurements and nested recording structure. Linear and quadratic fixed-effect coefficients were extracted for each PC. A single population-level depth effect magnitude was computed as the variance-weighted euclidean norm across PCs:

$$M = \sqrt{\sum_k w_k \beta_{k,\text{lin}}^2 + \sum_k w_k \beta_{k,\text{quad}}^2}$$

bioRxiv preprint doi: https://doi.org/10.64898/2026.10.21.760654; this version posted October 22, 2026. The copyright holder for this preprint (which was not certified by peer review) is the author/funder, who has granted bioRxiv a license to display the preprint in perpetuity. It is made available under a CC-BY 4.0 International license.

Where w_k is the variance explained by PC *k*. Statistical significance was assessed using a nonparametric permutation test. Depth values were randomly permuted within-channel, preserving channel-specific depth distributions while breaking the mapping between depth and neural activity. For each permutation, the full PCA–LME procedure was repeated and a null distribution of population magnitudes was constructed. The p-value was computed as the proportion of null magnitudes greater than or equal to the observed value.

Pitch tuning and acoustic feature GLM

For each neuron, for all segmented syllables, a 'harmonic index' was calculated every 2 ms by multiplying the harmonic ratio (MATLAB 2022a Audio Toolbox, Mathworks) by a Fundamental Index (FI), defined as the prominence of the fundamental frequency estimated from autocorrelation-based pitch tracking. Pitch, entropy, amplitude, and frequency modulation was computed using Sound Analysis Pro (SAP) (15) in 2 ms bins, and neural spikes were counted in the time window -30 to -5ms for each bin. Every bin with HR x FI > 0.6 was included in the pitch analysis. Relative contributions of the acoustic features to neuronal spiking was assessed using a Poisson generalized linear model (GLM) with an exponential link function. GLM significance ($p<0.01$) for each model was calculated using the likelihood ratio test statistic and shuffled permutations as described. Relative contribution of each predictor was calculated using McFadden's R2:

$$\frac{1-\frac{MR_{p,i}^2}{MR_f^2}}{\sum_{j=1}^{4}\left(1-\frac{MR_{p,j}^2}{MR_f^2}\right)}$$

Where $MR^2_{p,i}$ is McFadden's R2 for the partial model with the i'th predictor excluded and MR^2_f corresponds to the full model. The above analyses precisely replicate procedures described in a previous paper linking AAC spiking to acoustic features (30).

Nonlinear encoding models of vocal and movement contributions to neural activity

For each neuron with at least 20 headbob renditions, we modeled mean firing rate in sliding 50 ms windows as a function of vocal features, movement features, or both. Spectrograms spanning each headbob were processed using a 50 ms window (3 ms step) and passed through a trained autoencoder to obtain a 32-dimensional latent representation of vocal acoustics (*20*). In parallel, movement features were extracted within the same windows from skull-mounted accelerometers. Velocities along dorsal–ventral and anterior–posterior axes were estimated by cumulative trapezoidal integration followed by median filtering. For each window, velocity values at the beginning and end of the window were computed along both axes. Headbob phase at the beginning and end of the window, as well as absolute and relative time within the headbob bout, were included, yielding eight movement features per window. To capture nonlinear interactions between vocal and kinematic variables, we used gradient-boosted regression trees (XGBoost) (*26*, *27*). For each neuron, an ensemble of 200 trees (maximum depth 10) was trained with L1 ($\alpha = 0.1$) and L2 ($\lambda = 5$) regularization. Models were evaluated using grouped k-fold cross-validation, in which 20 s recording files served as independent groups, ensuring that all time bins from a given file were held out together to prevent inflation of performance estimates due to temporal autocorrelation (*49,50*). Model performance was

bioRxiv preprint doi: https://doi.org/10.64898/2026.10.21.760654; this version posted October 22, 2026. The copyright holder for this preprint (which was not certified by peer review) is the author/funder, who has granted bioRxiv a license to display the preprint in perpetuity. It is made available under a CC-BY 4.0 International license.

quantified as reduction in mean squared error relative to a null model predicting the mean firing rate of the training data. For each held-out file, improvement relative to the null was computed. Statistical significance was assessed using a one-sided Wilcoxon signed-rank test across files. To dissociate vocal and movement contributions, partial models using only vocal or only movement predictors were compared to the full model. Improvement of the full model over each partial model was evaluated using the same nonparametric test across held-out files. Neurons were classified as mixed if the full model significantly outperformed both partial models, indicating that joint inclusion of acoustic and kinematic features was required to explain spiking activity.

bioRxiv preprint doi: https://doi.org/10.64898/2026.10.21.760654; this version posted October 22, 2026. The copyright holder for this preprint (which was not certified by peer review) is the author/funder, who has granted bioRxiv a license to display the preprint in perpetuity. It is made available under a CC-BY 4.0 International license.

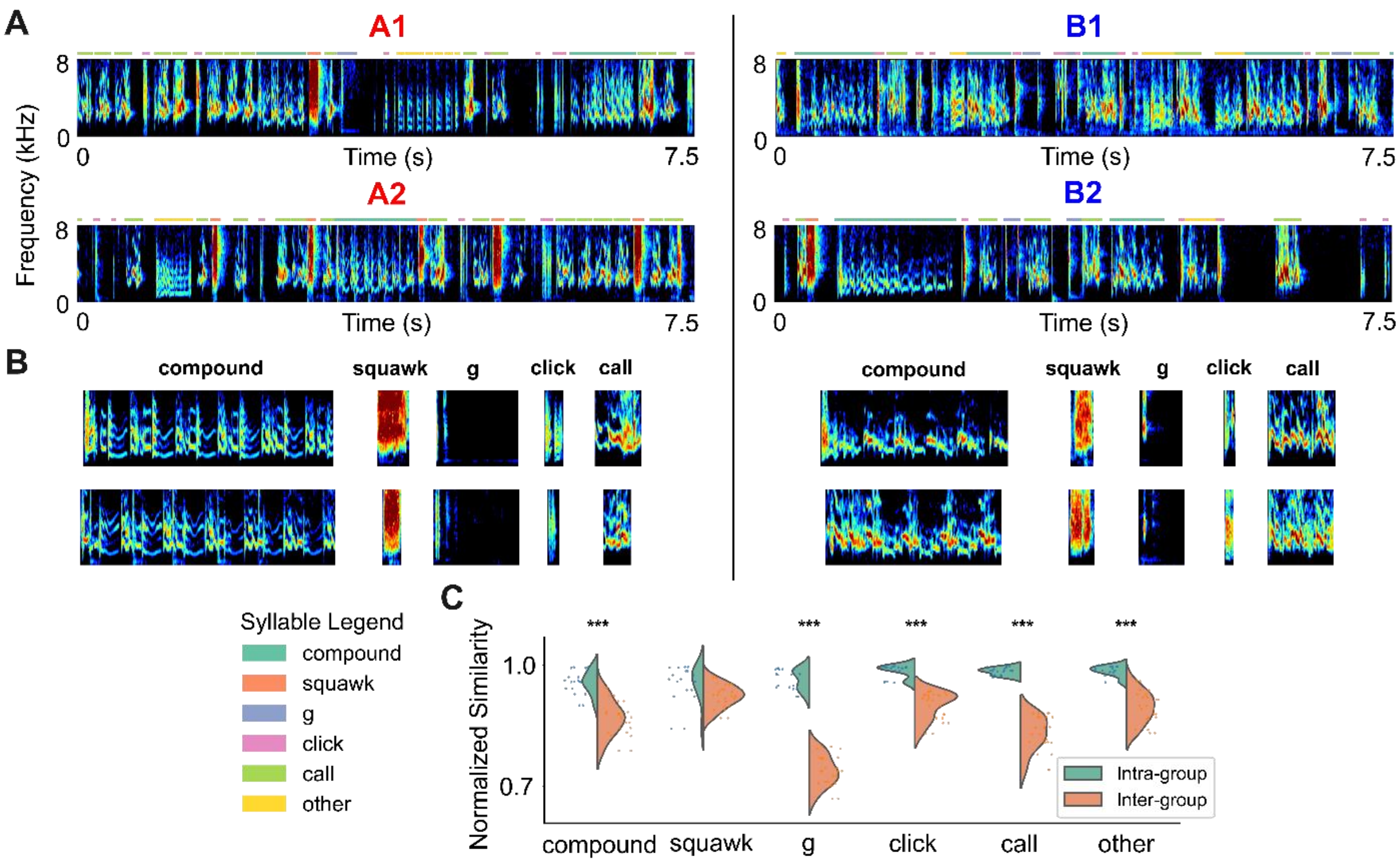

Fig. S1. Conventional syllable segmentation and similarity scoring methods confirm imitation by cagemates.

(**A**) Example warble bouts from representative birds in Cohort A (A1, A2) and Cohort B (B1, B2), color-coded by syllable category. (**B**) Representative spectrograms for each syllable type: Compound (long duration syllables with complex intrasyllabic repetitive acoustic structure); Squawk (brief broadband note); 'G' (click followed seamlessly by low frequency tail); Click (broadband note <50 ms); Call (contact call inserted into warble song). 'Other' was a category of other highly variable syllables unrecognizable to human eye or WhisperSeg. (**C**) Normalized similarity distributions comparing intra- versus inter-group pairs for each syllable class (***$p < 0.001$, two-sample t-test), indicating stronger convergence within cohorts.

bioRxiv preprint doi: https://doi.org/10.64898/2026.10.21.760654; this version posted October 22, 2026. The copyright holder for this preprint (which was not certified by peer review) is the author/funder, who has granted bioRxiv a license to display the preprint in perpetuity. It is made available under a CC-BY 4.0 International license.

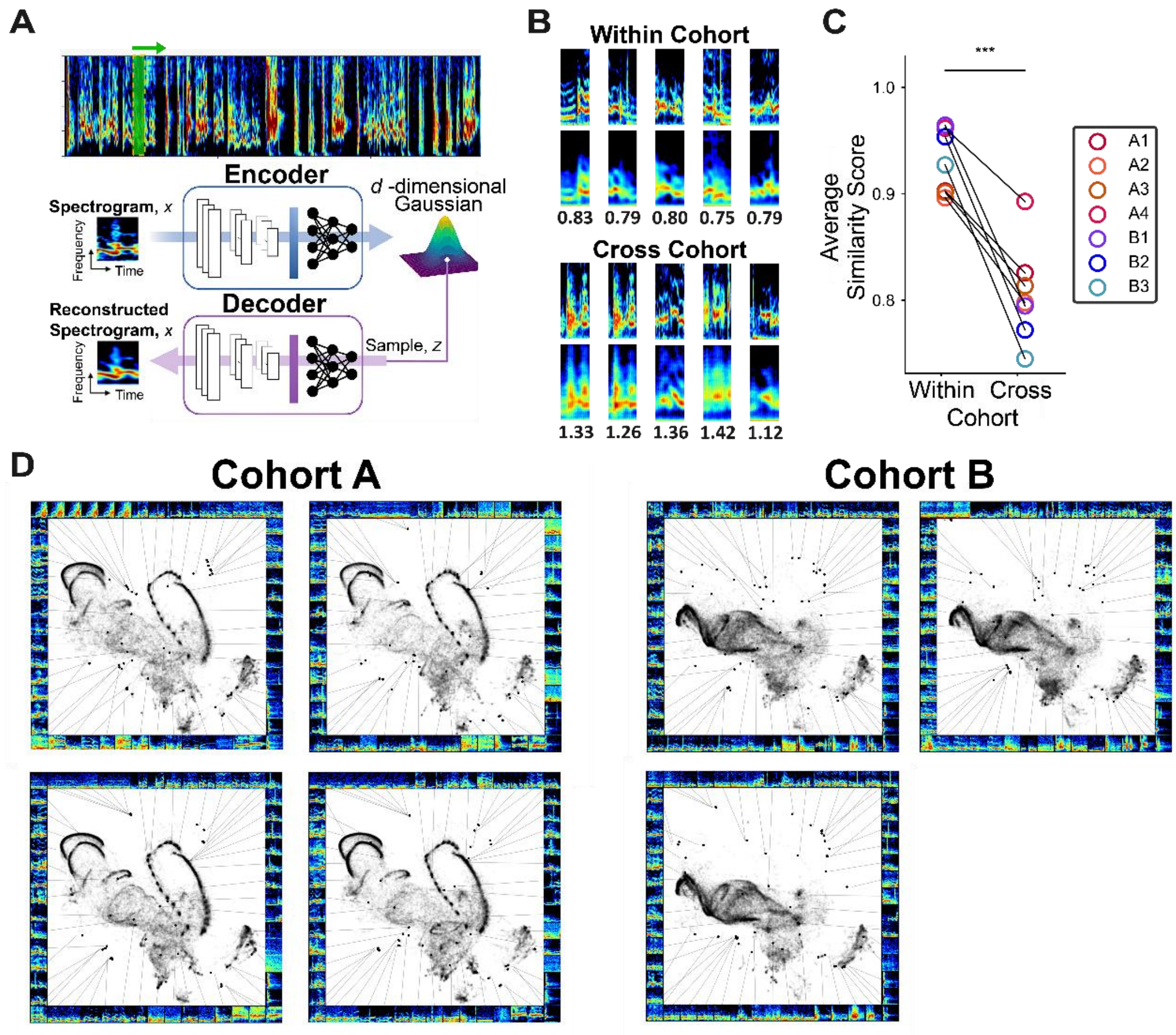

Fig. S2. VAE latent space analyses quantify warble imitation.

(**A**) A "shotgun" variational autoencoder (VAE) approach in which sliding 80-ms spectrogram windows (5-ms step size) are embedded into a 32-dimensional latent space. (**B**) Example reconstructions and reconstruction error for a VAE trained on bird A1, evaluated on held-out A1 warbles (top) or warbles from bird B1 (bottom). Reconstruction error is lower for within-bird evaluation and higher for cross-cohort evaluation, reflecting acoustic dissimilarity. (**C**) Acoustic similarity was quantified using a reconstruction-based similarity score, defined as the ratio of mean-squared reconstruction error (MSE) on the training data to the MSE on an evaluation dataset. Similarity scores approach 1 for acoustically similar warbles and decrease as vocal structure diverges; scores were significantly higher within cohorts than across cohorts. (**D**) Two-dimensional UMAP projections of the VAE latent space for birds A1–A4 and B1–B3. Warble manifolds are highly similar within cohorts but distinct across cohorts, revealing socially learned, colony-specific acoustic structure.

bioRxiv preprint doi: https://doi.org/10.64898/2026.10.21.760654; this version posted October 22, 2026. The copyright holder for this preprint (which was not certified by peer review) is the author/funder, who has granted bioRxiv a license to display the preprint in perpetuity. It is made available under a CC-BY 4.0 International license.

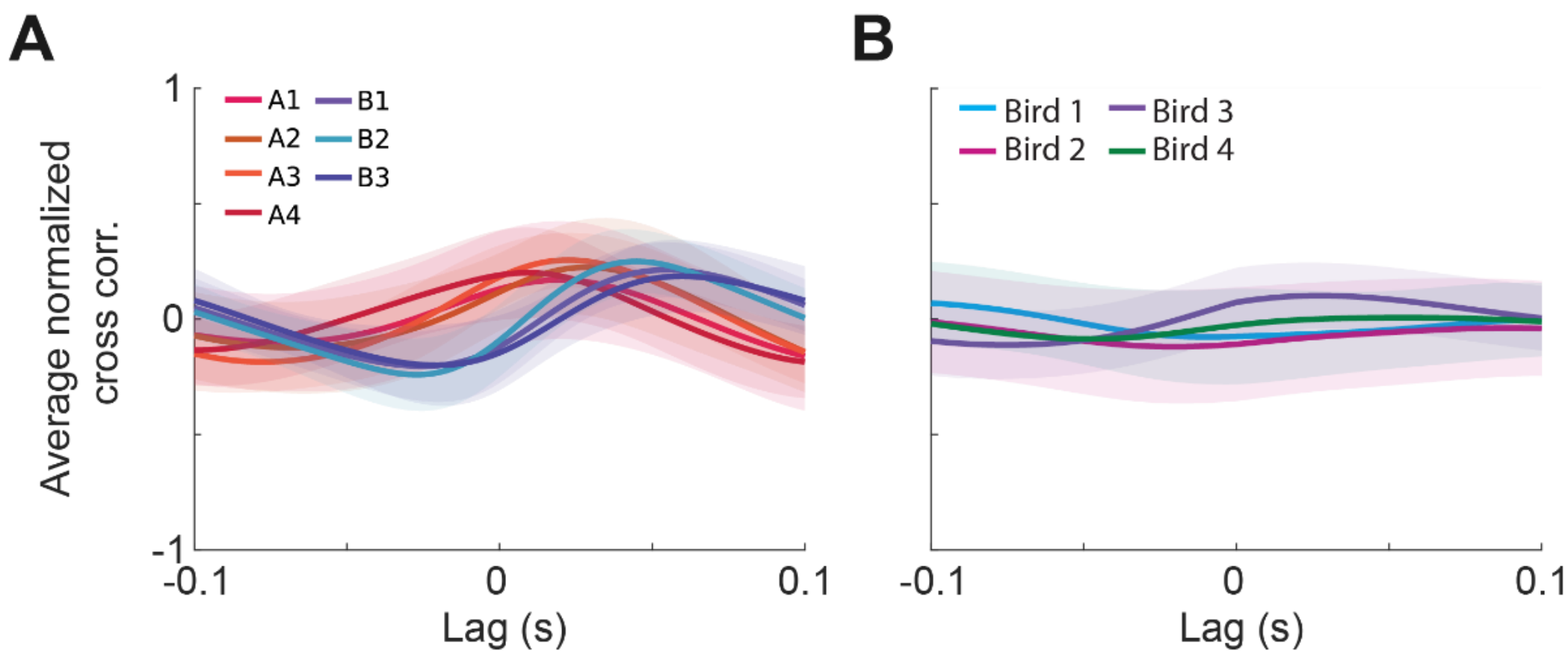

Fig. S3. The relationship between head velocity and sound amplitude was colony-specific. **(A)** Average +/1 STD cross correlation between sound amplitude and head velocity. A permutation test showed that cohort A and B birds exhibited significantly distinct curve shapes ($p<0.01$). **(B)** Data plotted as in A for the 4 birds examined in the electrophysiology experiments, which had no social contact with the colony A or B birds.

bioRxiv preprint doi: https://doi.org/10.64898/2026.10.21.760654; this version posted October 22, 2026. The copyright holder for this preprint (which was not certified by peer review) is the author/funder, who has granted bioRxiv a license to display the preprint in perpetuity. It is made available under a CC-BY 4.0 International license.

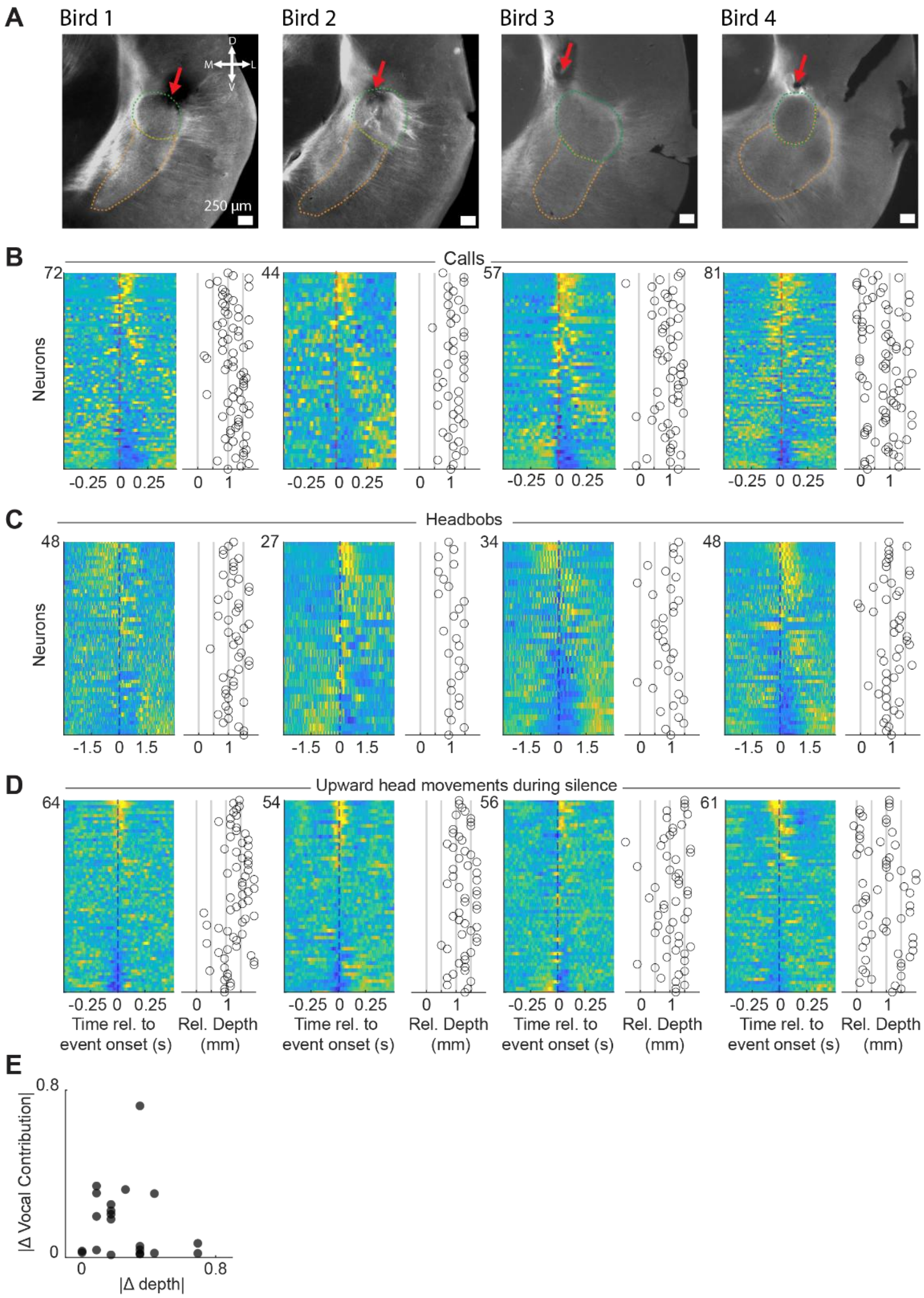

Fig. S4. Vocal and gestural representations are spatially overlapping in AAC.

(**A**) Histological confirmations of cannula position (red arrows), dorsal to AAC core, from which electrodes were advanced. Green and orange dotted lines denote boundaries of dorsal core and ventral shell, respectively. (**B**) Call-aligned z-scored firing rates of 72 AAC neurons sorted by the first principal component (PC1) of the individual bird population call-aligned activity; scatter plots show the recording depth of each neuron (row-aligned), relative to the estimated start of AAc core (mm). (**C**) Data plotted as in B for headbob aligned activity. In all birds, call- and headbob-locked neural representations were found spatially intermingled at all sites. (**D**) Data plotted as in B,C for activity aligned to upward movements of the head during silent times. (**E**) Within bird, within channel comparisons of absolute difference in recording depth vs absolute difference in the vocal contribution metric shown in Fig. 4G.

bioRxiv preprint doi: https://doi.org/10.64898/2026.10.21.760654; this version posted October 22, 2026. The copyright holder for this preprint (which was not certified by peer review) is the author/funder, who has granted bioRxiv a license to display the preprint in perpetuity. It is made available under a CC-BY 4.0 International license.

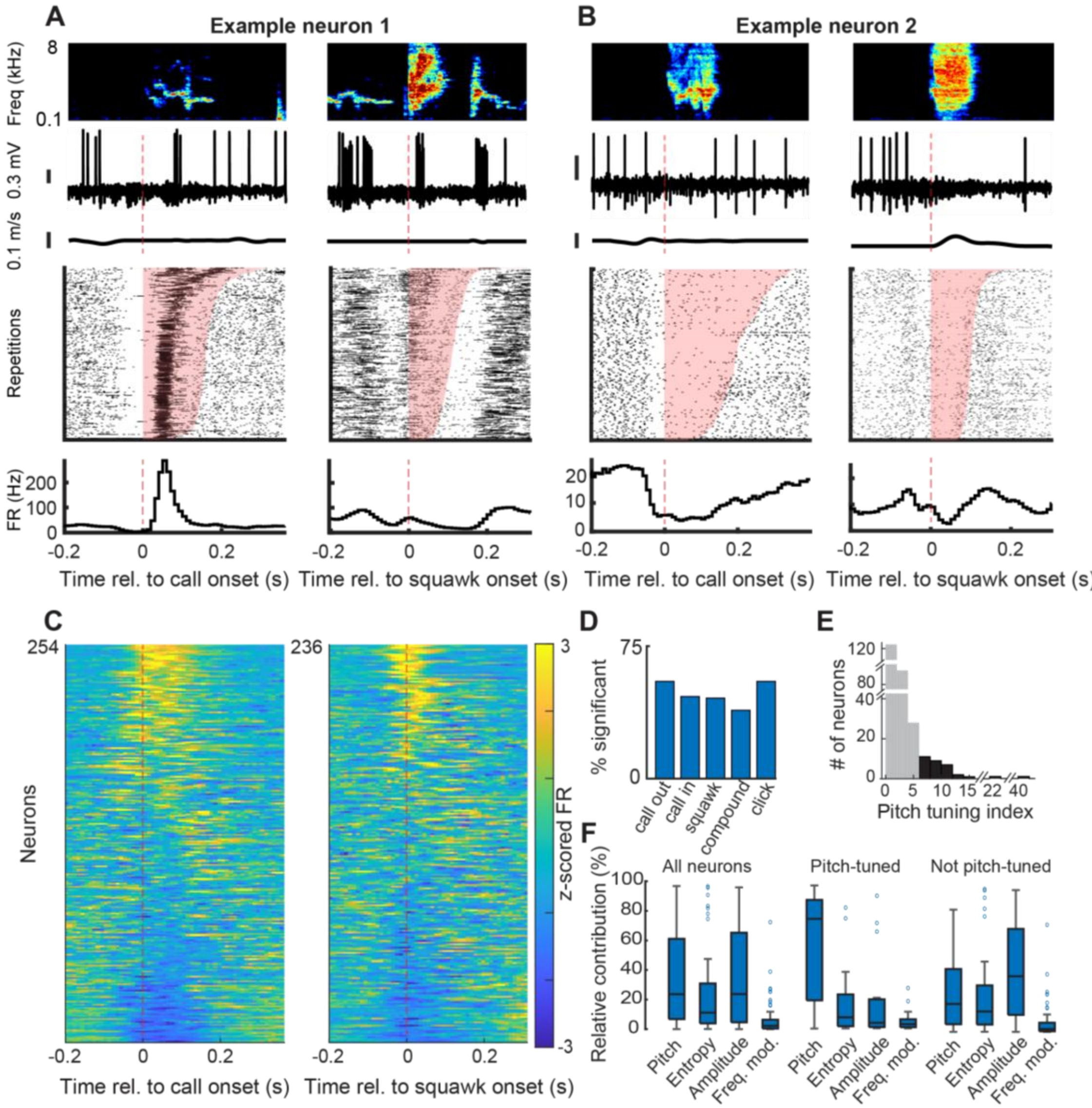

Fig. S5. Acoustic and segmentation-based analyses confirm vocalization-locked activity of AAC neurons.

(**A-B**) Call (left) and squawk (right) aligned activity of two example AAC neurons. (**C**) Population call- and squawk-aligned activity for 254 and 236 AAC neurons, respectively, sorted by the first principal component (PC1) score of the call- or squawk aligned population response. (**D**) Proportion of neurons exhibiting significant firing-rate maxima or minima for different vocalization types. (**E**) Distribution of pitch-tuning index values. Gray bars denote non-pitch-tuned neurons (index < 6); black bars denote pitch-tuned neurons (index ≥ 6). Axes are broken to enhance visualization. (**F**) Relative contributions of different acoustic features to firing-rate prediction in a generalized linear model (GLM). These findings confirm a recent study by the Long lab (*25*), including neuronal tuning to pitch, showing that the gestural signals we observe are in the same population of neurons.

bioRxiv preprint doi: https://doi.org/10.64898/2026.10.21.760654; this version posted October 22, 2026. The copyright holder for this preprint (which was not certified by peer review) is the author/funder, who has granted bioRxiv a license to display the preprint in perpetuity. It is made available under a CC-BY 4.0 International license.

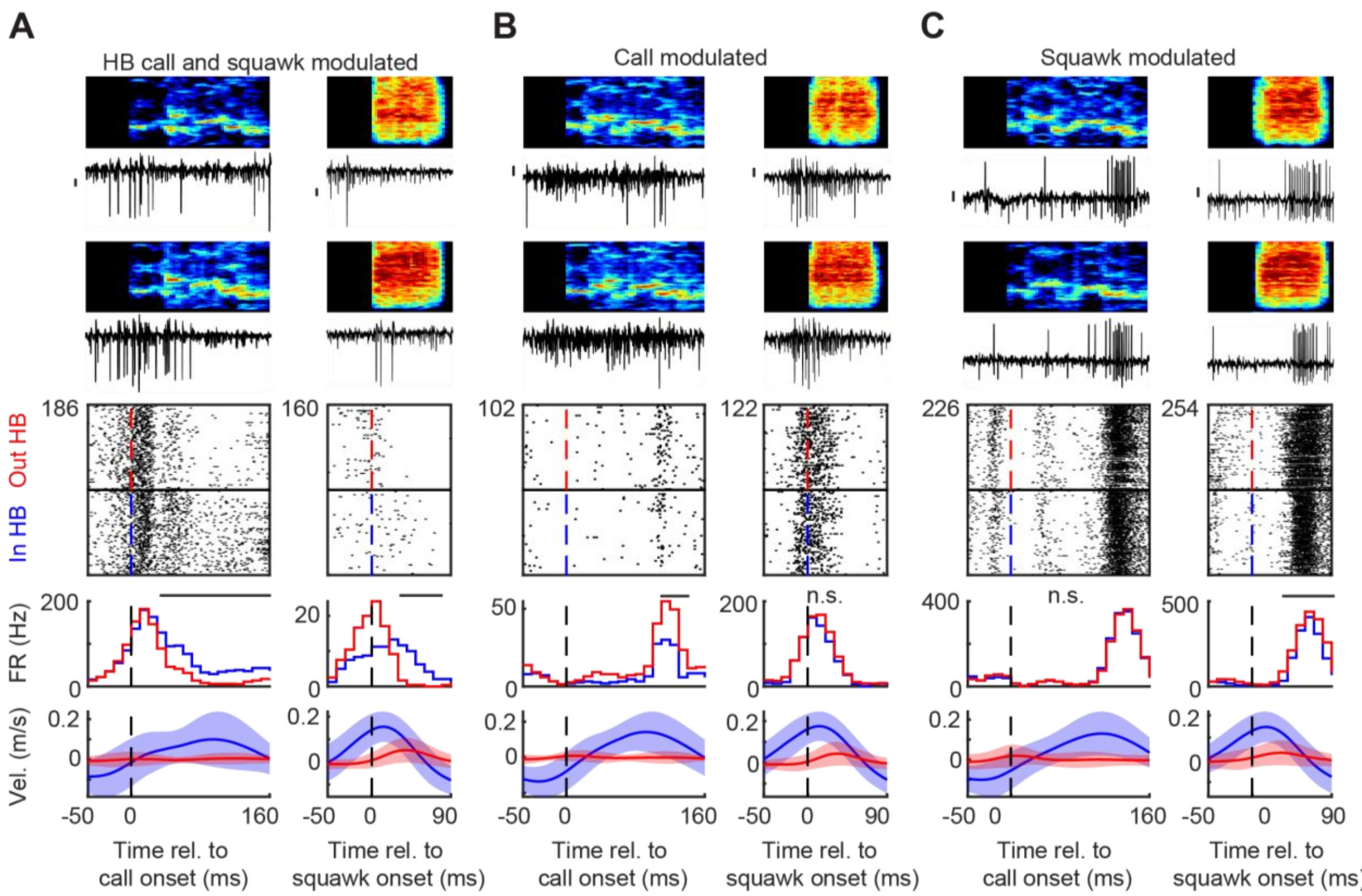

Fig. S6. Headbobs can modulate syllable-locked AAC activity even when syllable acoustics are matched.

(**A**) Neuron showing differential activity for calls produced outside versus during headbob (HB) bouts. Left column (top to bottom): spectrograms and spiking for nearest-neighbor call renditions outside and inside HB; call-aligned rasters (outside HB, top; inside HB, bottom); rate histogram for outside (red) and inside (blue) HB trials, and call-aligned head velocity traces (mean±SD.). Right column: same analyses for squawk syllables. Neurons were classified as HB-modulated if ≥3 consecutive 10-ms bins differed significantly between conditions (nonparametric shuffle test, $p < 0.001$, black bars). (**B**) Neuron exhibiting significant HB-dependent modulation for calls only. (**C**) Neuron exhibiting HB-dependent modulation for squawks only.

bioRxiv preprint doi: https://doi.org/10.64898/2026.10.21.760654; this version posted October 22, 2026. The copyright holder for this preprint (which was not certified by peer review) is the author/funder, who has granted bioRxiv a license to display the preprint in perpetuity. It is made available under a CC-BY 4.0 International license.

Bird	Cohort	Duration (s)	Amplitude	Frequency (Hz)	# headbobs
A1	A	0.70 ± 0.28	2.82 ± 2.08	5.70 ± 4.19	3.42 ± 1.38
A2	A	1.26 ± 0.42	3.07 ± 2.66	4.92 ± 2.01	5.61 ± 1.90
A3	A	1.33 ± 0.55	2.82 ± 2.54	5.82 ± 3.70	6.64 ± 2.79
A4	A	1.13 ± 0.33	2.49 ± 1.94	4.88 ± 1.81	5.04 ± 1.53
B1	B	1.34 ± 0.55	2.85 ± 2.09	4.70 ± 1.31	5.95 ± 2.39
B2	B	1.12 ± 0.45	2.77 ± 2.54	4.74 ± 1.23	5.01 ± 1.99
B3	B	1.10 ± 0.42	2.76 ± 2.00	4.54 ± 1.03	4.78 ± 1.79

Table S1. Headbob kinematic measures by bird.
Values represent per-bird medians across headbob bouts for bout duration, amplitude, bob frequency, and number of bobs per bout. Birds are grouped by cohort (A or B)..

Movies S1 – S8.
Example video (0.5× speed) of a budgerigar performing headbobs toward a one way mirror. Shown alongside the video are the vocal spectrogram (top), head velocity (middle), and projections of the latent acoustic space with relative time in the headbob sequence and cycle phase overlaid (bottom right).
